# Novel miRNA markers for the diagnosis and prognosis of endometrial cancer

**DOI:** 10.1111/jcmm.15111

**Published:** 2020-03-09

**Authors:** Qian Wang, Kai Xu, Yu Tong, Xianning Dai, Teng Xu, Danna He, Jianchao Ying

**Affiliations:** ^1^ Department of Clinical Laboratory Wenzhou People's Hospital The Third Clinical Institute Affiliated to Wenzhou Medical University Wenzhou China; ^2^ Department of Cardiology Institute of Translational Medicine Baotou Central Hospital Baotou China; ^3^ Central Laboratory Institute of Emergency Medicine The First Affiliated Hospital of Wenzhou Medical University Wenzhou China

**Keywords:** diagnostic classifier, endometrial cancer, microRNA, molecular biomarker, prognostic model

## Abstract

As endometrial cancer (EC) is a major threat to female health worldwide, the ability to provide an accurate diagnosis and prognosis of EC is promising to improve its treatment guidance. Since the discovery of miRNAs, it has been realized that miRNAs are associated with every cell function, including malignant transformation and metastasis. This study aimed to explore diagnostic and prognostic miRNA markers of EC. In this study, differential analysis and machine learning were performed, followed by correlation analysis of miRNA‐mRNA based on the miRNA and mRNA expression data. Nine miRNAs were identified as diagnostic markers, and a diagnostic classifier was established to distinguish between EC and normal endometrium tissue with overall correct rates >95%. Five specific prognostic miRNA markers were selected to construct a prognostic model, which was confirmed more effective in identifying EC patients at high risk of mortality compared with the FIGO staging system. This study demonstrates that the expression patterns of miRNAs may hold promise for becoming diagnostic and prognostic biomarkers and novel therapeutic targets for EC.

## 1 INTRODUCTION

Endometrial cancer (EC), one of most common gynaecological malignant tumours, threatens the female health worldwide, especially in developed countries.^1^ According to estimated data, more than 63 230 new EC cases and 11 350 EC deaths are projected to occur in the United States in 2018.^2^ Current diagnoses for uterine corpus tumours mainly depend on clinical and histological features. However, 15%‐20% of these tumours still have a high risk of recurrence and even further deterioration. Although some molecular classification systems or biomarkers have been proposed to complement the current risk stratification approaches,^3^, ^4^, ^5^, ^6^ none of them have been widely applied or become a standard of care in clinical practice. Thus, the identification of effective and reliable biomarkers for the early diagnosis and prognosis of EC remains a significant clinical challenge.

MicroRNAs (miRNAs), a class of short noncoding RNAs with a length of 19‐24 nucleotides, were demonstrated to work as regulators of gene expression by antisense complementarity or complementarity into specific mRNAs.^7^ Many studies have shown that multiple miRNAs are aberrantly expressed in various tumours and are involved in tumorigenesis and progression as oncogenes or tumour suppressor genes.^8^, ^9^ A number of studies have demonstrated that miRNA expression is associated with cell proliferation, metastasis, invasion and response to therapy.^10^, ^11^ Moreover, miRNAs act as highly specific, sensitive and stable molecules, making them potential markers for diagnosing specific cancers and their progression.^12^ With the rapid development of various miRNA sequencing technologies, especially next‐generation sequencing technology, miRNA signatures in EC could be used to distinguish EC from normal counterparts and may shed light on the molecular diagnosis and prognosis of EC. Previous studies have tried to determine the correlation between miRNAs and EC. For instance, has‐mir‐337‐3p, let‐7b and miR135a were indicated to have the potential to serve as noninvasive biomarkers,^13^ and the abnormal expression patterns of several miRNAs were suggested to have a strong association with carcinogenesis in the early stages. Previous research indicated that the expression pattern of miRNAs in EC can be correlated with their respective target mRNAs, inferred from an increase in the expression of several oncogenic proteins such as ERBB2, EGFR, EPHA2, BAX, GNA12, GNA13 and JUN, further substantiating that the combination of miRNAs with target mRNAs play an important role in carcinogenesis.^1^ Thus, miRNAs have the potential to become novel noninvasive biomarkers measured as diagnostic and prognostic indicators of EC to guide surgical therapies and promote the understanding of the carcinogenesis of EC.

In this study, we aimed to identify potential diagnostic and prognostic miRNA biomarkers in EC. Using miRNA expression data in EC patients, we constructed a novel diagnostic miRNA classifier for distinguishing between EC and normal endometrium tissues, and a prognostic miRNA model for survival prediction in EC patients. Finally, these two novel models were validated and evaluated, respectively, indicating their diagnostic or prognostic values for EC.

## 2 MATERIALS AND METHODS

### 2.1 Data collection and preprocessing

The miRNA expression and mRNA expression data of patients with endometrioid endometrial adenocarcinoma were obtained from the public databases including The Cancer Genome Atlas Program (TCGA, https://portal.gdc.cancer.gov/) and Gene Expression Omnibus Datasets (GEO, https://www.ncbi.nlm.nih.gov/geo/). Dealing with TCGA data, the patients whose clinical information and follow‐up data were not complete were excluded, whereas only patients with fully characterized mRNA and miRNA profile data were collected in our study (Table S1). Moreover, the study types were limited to RNA‐Seq based on IlluminaHiSeq/IlluminaGA platforms (processed by HTSeq). According to the inclusion criteria, a total of 441 samples for miRNA expression data and 419 samples for mRNA expression data were enrolled in further analysis (Table S2). The entire cohort from TCGA database (N = 419) was randomly divided into training and testing cohorts according to the ratio of 2 to 1 (Table S2). Of note, the miRNAs/mRNAs with reads count > 1 that did not exist across 50% of samples in the training cohort were excluded. The normalization of expression levels was performed using the DESeq2 package^14^ in R, and batch correction was processed by limma package. miRNA expression microarray data (from GEO database) were normalized and transformed to expression values by using affy package.

### 2.2 Differential expression analysis of miRNAs and genes

Differential expression between EC tissues and normal counterparts in the training cohort was assessed using the DESeq2 package, and the adjusted *P* value was calculated afterwards. The differentially expressed miRNAs and genes were then screened with the filtering criteria of an adjusted *P* value < .001. Mann‐Whitney *U* test implemented in SciPy package was conducted to examine the differential expression level of miRNA marker in the testing cohort.

### 2.3 Identification of diagnostic miRNA markers

Least absolute shrinkage and selection operator (LASSO), a method of automatic variable selection in high dimensional data, was used for the selection of diagnostic miRNAs. As previously described, the tuning parameters were determined according to the expected generalization error estimated from 10‐fold cross‐validation.^6^ Unsupervised hierarchical clustering of the expression pattern of these diagnostic miRNA markers was conducted using the pheatmap package. Based on the expression level of these miRNA markers, the diagnostic classifier was constructed by implementing LASSO method under a binomial distribution. Receiver operating characteristic (ROC) curves and confusion matrices were subsequently applied to evaluate the prediction accuracy of the miRNA markers and diagnostic classifier. The best cut‐off values in ROC curves were obtained for distinguishing EC and normal endometrium tissues in a confusion table.

### 2.4 Identification of prognostic miRNA markers

As a prescreening procedure, the univariate Cox regression analysis was performed to identify miRNAs/genes associated with survival. A variable hunting method implemented in the randomForestSRC package was employed to screen candidate prognostic markers. Subsequently, multivariate Cox regression was applied to construct a prognostic model and remove any miRNAs that might not be independent factors in the model. For the gene model devised by our previous work, the risk score for each patient was computed using the list of nine genes (*SLC16A1‐AS1*, *KDM4B*, *MAP2K5*, *SYP*, *MPP1*, *DLX4*, *BOLA3‐AS1*, *HOMEZ* and *STAP2*).^15^ For the prognostic model proposed by Wang et al,^16^ six genes (*PCSK4*, *IHH*, *CTSW*, *LRRC8D*, *TNFRSF18* and *CDKN2A*) were used to calculate the risk score for EC patient. To evaluate the ability of the prognostic model, the concordance index was calculated. In addition, the survivalROC package was applied to calculate time‐dependent ROC curve from censored survival data and compute the value of the area under the curve (AUC value). Moreover, the ability of the model to predict outcome at three and 5 years was assessed, respectively. According to the maximum Youden index in the ROC curve, the best cut‐off point was obtained to divide patients into different prognostic groups. Kaplan‐Meier curves were then plotted to evaluate the correlations between models and overall survival (OS). Meanwhile, hazard ratio (HR) and *P* values were computed by using the ‘survdiff’ function in the survival package. All aforementioned *P* values were two‐sided.

### 2.5 Correlation analysis of miRNA‐mRNA expression

miRNA‐mRNA regulation interactions were identified by two criteria. First, the pairwise correlation coefficients between differentially expressed miRNAs and genes were calculated by Pearson's correlation test. A *P* value less than .05 was considered to be statistically significant. Second, six miRNA‐target prediction tools/databases (miRWalk,^17^ miRDB, RNA22, miRanda, PICTAR2 and Targetscan) were employed to predict target genes regulated by miRNA markers. The predicted miRNA‐target pairs were screened out by no less than four algorithms, except hsa‐miR‐7706, which was screened out by no less than three. Additionally, the miRNA‐target pairs verified by experiments in the miRWalk database were also included. All the miRNA‐target pairs were finally determined, which were not only negatively correlated but also predicted by algorithms (or verified by experiment). Then, the miRNA‐target regulatory network was constructed, which was visualized using Cytoscape program. ClusterProfiler^18^ package was used to perform over‐representation analysis on Kyoto Encyclopedia of Genes and Genomes (KEGG) pathways associated with the target genes regulated by miRNAs. The tool took the target gene list and the background gene list of whole human as input and conducted statistical enrichment analysis using hypergeometric testing. The pathways were considered significantly enriched when their *P* values were smaller than .05.

## 3 RESULTS

### 3.1 Differentially expressed miRNAs in EC

**Figure 1:**
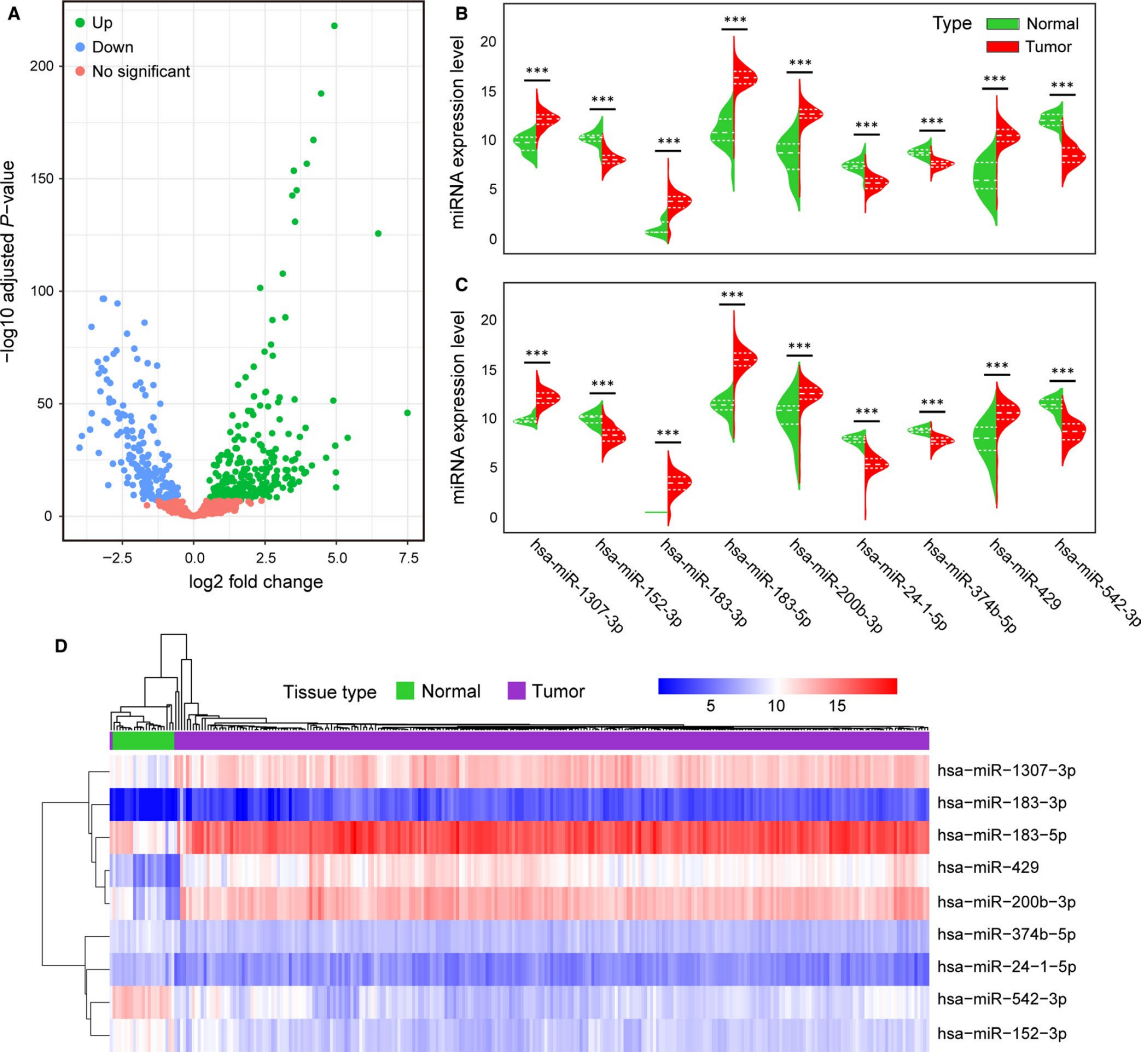
Identification of miRNA markers for diagnosis of EC. A, Differential expression analysis of miRNAs in the training cohort. B, C, The distributions of the miRNA expression data are represented by violin plots, and the dashed lines indicate the quartiles. B, The miRNA expression of nine markers in 258 tissues and 21 normal endometrium tissues in the training cohort. Adjusted *P* values were obtained from differential expression analysis by DESeq2. C, The miRNA expression of nine markers in 129 tissues and 11 normal endometrium tissues in the testing cohort. *P* values were calculated by the Mann‐Whitney *U* test. (*P < .05, **P < .01, ***P < .001) (D) Unsupervised hierarchical clustering and heatmap of the training cohort based on the expression profiles of the nine miRNA markers selected

The training cohort, which comprised EC (N = 258) and normal endometrium (N = 21), was included in this analysis. By performing differential expression analyses, there were 417 differentially expressed miRNAs with adjusted *P* value < .001 between EC and normal endometrium tissues, including 238 up‐regulated miRNAs and 179 down‐regulated miRNAs (Figure 1A, Table S3). The top ten up‐regulated miRNAs were hsa‐miR‐183‐5p, hsa‐miR‐96‐5p, hsa‐miR‐182‐5p, hsa‐miR‐429, hsa‐miR‐200b‐3p, hsa‐miR‐200a‐5p, hsa‐miR‐200b‐5p, hsa‐miR‐200a‐3p, hsa‐miR‐205‐5p and hsa‐miR‐141‐3p, whereas the top ten down‐regulated miRNAs were hsa‐miR‐542‐5p, hsa‐miR‐542‐3p, hsa‐miR‐101‐3p, hsa‐miR‐28‐5p, hsa‐miR‐424‐3p, hsa‐miR‐140‐3p, hsa‐miR‐152‐3p, hsa‐miR‐450b‐5p, hsa‐miR‐450a‐5p and hsa‐miR‐139‐5p.

To further understand the potential biological function of these differentially expressed miRNAs in EC, functional enrichment analysis of these miRNAs was carried out. As a result, these miRNAs were found to be significantly related to pathways in cancer, the cell cycle, focal adhesion, the Wnt signalling pathway, cell adhesion molecules etc (Table S4, *P* < .05).

### 3.2 Diagnostic miRNA markers for EC

By using LASSO, the number of these differentially expressed miRNAs was reduced to screen optimal signatures for diagnosis of EC. A panel of nine miRNAs was ultimately selected as diagnostic markers. In both the training and testing cohorts, the expression levels of hsa‐miR‐1307‐3p, hsa‐miR‐183‐3p, hsa‐miR‐183‐5p, hsa‐miR‐200b‐3p and hsa‐miR‐429 were significantly up‐regulated in EC tissues whereas the expression levels of hsa‐miR‐152‐3p, hsa‐miR‐24‐1‐5p, hsa‐miR‐374b‐5p and hsa‐miR‐542‐3p were down‐regulated in EC tissues (*P* < .001, Figure 1B,C). Unsupervised hierarchical clustering of training cohort samples was performed based on the expression pattern of these miRNA markers, and the heatmap demonstrated that most of the same types of tissues could cluster together (Figure 1D). These results suggest that these miRNAs might serve as diagnostic markers to distinguish EC from normal endometrium tissues.

**Figure 2:**
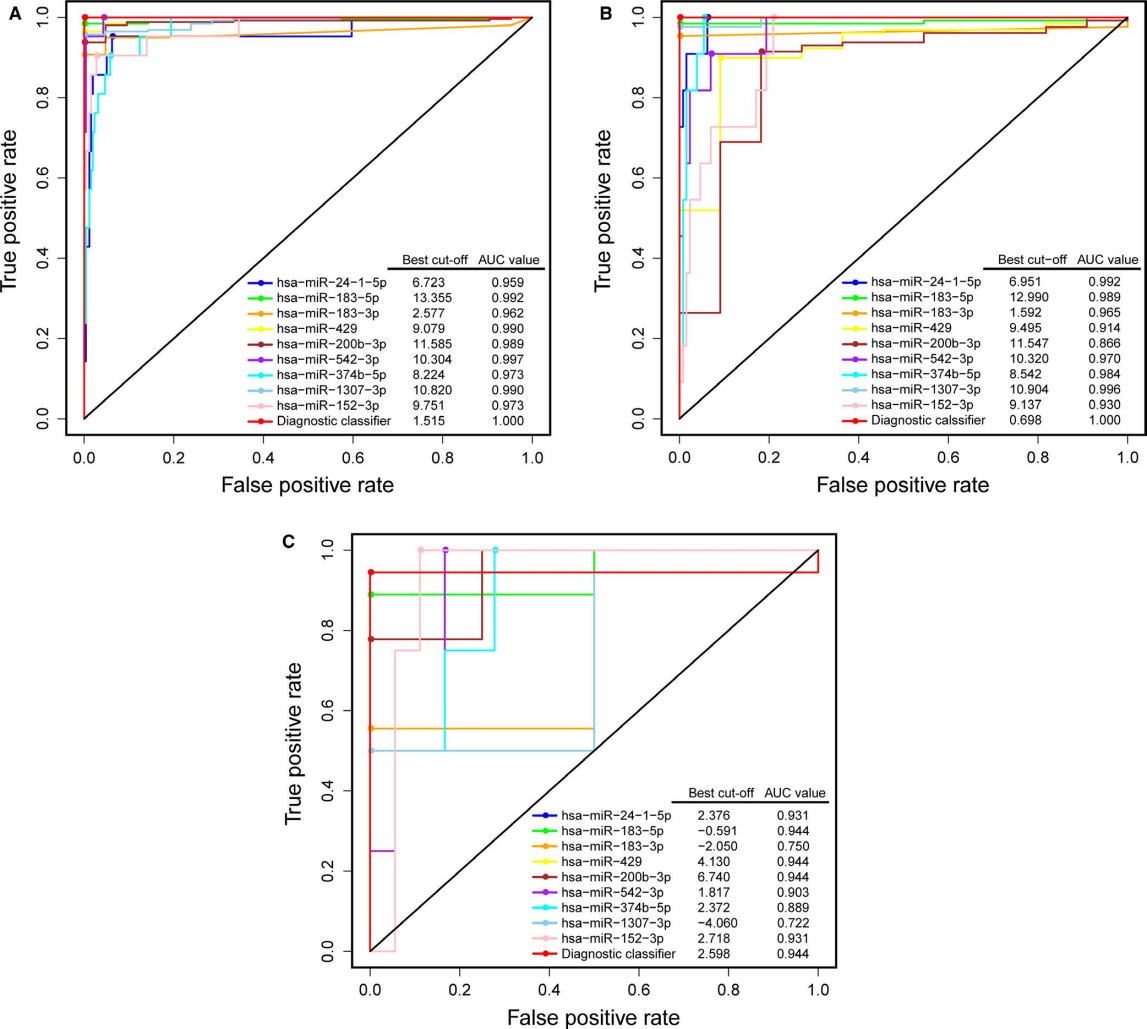
Performance of the miRNA diagnostic classifiers in tissue prediction. ROC curves of diagnostic classifiers built on each miRNA and all nine miRNA markers together in the training (A), testing (B) and GSE35794 (C) cohorts. The best cut‐off values in ROC curves were obtained to distinguish between EC and normal endometrium tissues

Subsequently, a combined diagnostic classifier was established using these nine miRNA markers to identify the group members of the tissue samples (Table 1). In addition, ROC curves were plotted to compare the efficiency of these diagnostic classifiers built on one miRNA or all nine miRNA markers. In the training cohort, the AUC of the combined classifier was 1.000, followed by hsa‐miR‐542‐3p with an AUC of 0.997 and hsa‐miR‐183‐5p with an AUC of 0.992 (Figure 2A). In the testing cohort and an additional cohort (GSE35794), the AUC of the combined classifier was higher than or similar to other classifiers (Figure 2B,C). Therefore, it is clear that the combined diagnostic classifier showed better performance in tissue prediction than other classifiers built on a single miRNA marker. When the final diagnostic classifier (combined classifier) was applied to the training, testing and GSE35794 cohorts, we obtained overall correct diagnosis rates of 100%, 100% and 95.45%, respectively, which point to the potential utility of the combined classifier (Table 2).

**Table 1:** The diagnostic miRNA classifier constructed in this study

Marker	MIMATid	Coefficient	Role in carcinogenesis of EC
hsa‐miR‐542‐3p	MIMAT0003389	−1.057	Suppressor miRNA
hsa‐miR‐152‐3p	MIMAT0000438	−0.248	Suppressor miRNA
hsa‐miR‐24‐1‐5p	MIMAT0000079	−0.202	Suppressor miRNA
hsa‐miR‐374b‐5p	MIMAT0004955	−0.115	Suppressor miRNA
hsa‐miR‐183‐3p	MIMAT0004560	0.030	OncomiRNA
hsa‐miR‐200b‐3p	MIMAT0000318	0.035	OncomiRNA
hsa‐miR‐429	MIMAT0001536	0.050	OncomiRNA
hsa‐miR‐1307‐3p	MIMAT0005951	0.135	OncomiRNA
hsa‐miR‐183‐5p	MIMAT0000261	0.559	OncomiRNA

**Table 2:** Confusion table of the training, testing and GSE35794 cohorts using miRNA diagnostic classifier

	Training cohort (N = 279)			Testing cohort (N = 140)			GSE35794 (N = 22)		
	EC	NE	Total	EC	NE	Total	EC	NE	Total
EC	258	0		129	0		17	0	
NE	0	21		0	11		1	4	
Wrong tissue	0	0		0	0		1	0	
Total	258	21	279	129	11	140	18	4	22
Correct (%)	100.00	100.00	100.00	100.00	100.00	100.00	94.44	100.00	95.45

Note

The rows represent the predictions, and the columns represent the true values. The values of the table indicate the predicted/true number of a cancer type (represented by the row) in training, testing and GSE35794 cohorts, respectively. Wrong tissue represents the misclassified types of cancer or normal endometrium samples.

Abbreviations: EC, endometrial cancer; NE, normal endometrium.

### 3.3 Correlation of diagnostic miRNA markers and their regulated genes

**Figure 3:**
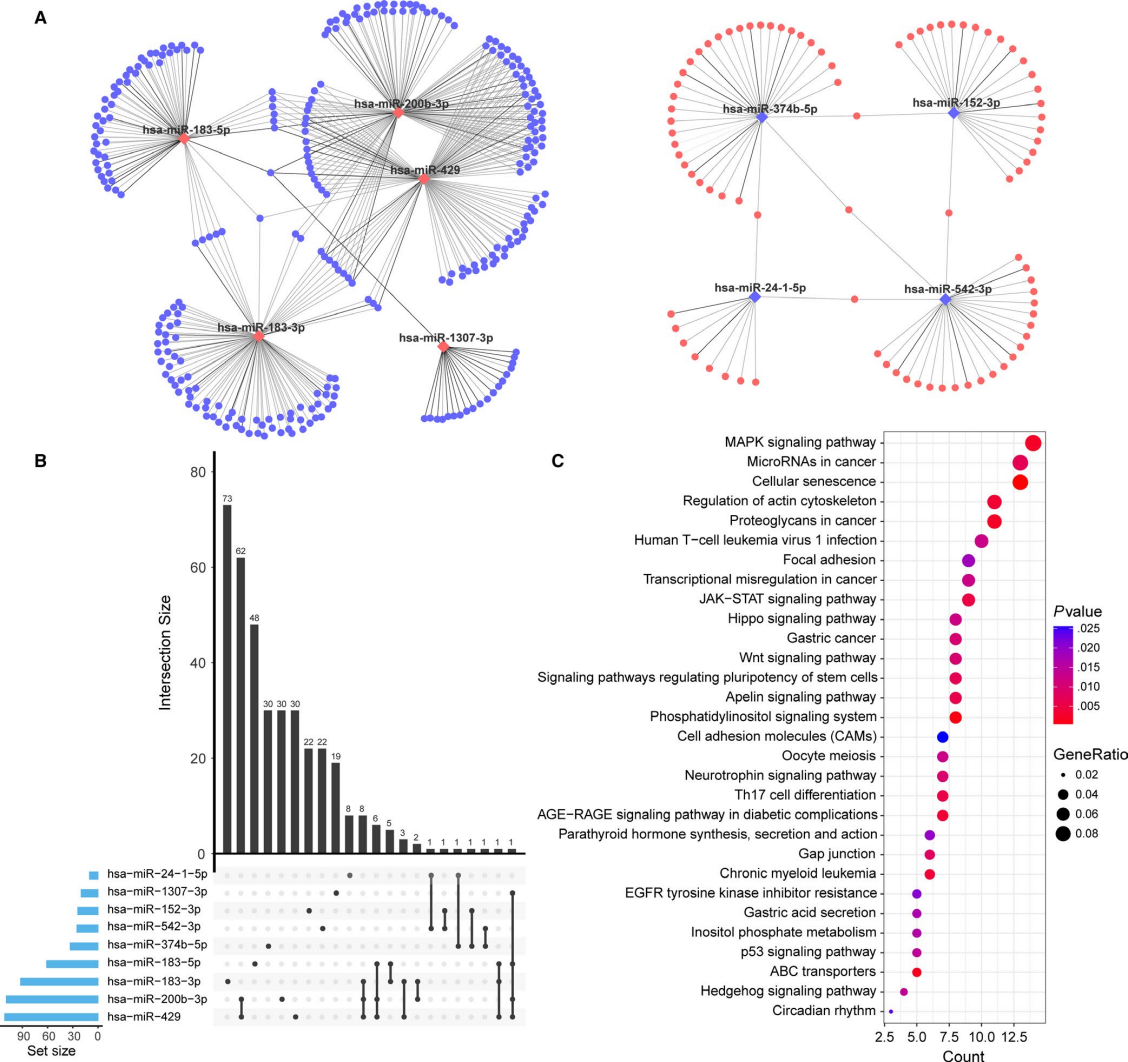
The regulatory network of the nine miRNA markers and target genes in EC and the enrichment analysis of these target genes. A, Cytoscape visualization of the 485 miRNA‐mRNA pairs. The diamonds and circles represent the miRNAs and target genes, respectively. Red and blue colours represent up‐regulation and down‐regulation, respectively. The grey edge indicates the verified miRNA‐target pairs, whereas the black edge indicates the predicted pairs. B, The UpSet plot of interactions between the differentially expressed genes regulated by the nine diagnostic miRNA markers. The plot was generated to visualize the intersecting sets between different genes and miRNA markers. One gene might be regulated by up to four miRNAs. C, KEGG functional enrichment analysis for 375 target genes regulated by the nine diagnostic miRNA markers. Only the top 30 significant pathways are displayed

To explore the potential interactions for these nine miRNA markers and their regulated genes, we integrated the miRNA and gene expression data of EC. A differential analysis between EC and normal endometrium tissues was conducted, and 6501 differentially expressed protein‐coding genes were identified with the cut‐off of an adjusted *P* < .001. By performing the miRNA‐mRNA expression correlation analysis, 485 miRNA‐mRNA pairs, which were inversely associated as well as predicted by algorithms or verified by experiment, were ultimately obtained (Table S5). Meanwhile, a miRNA‐mRNA regulation network was constructed, which showed that the expression level of 375 genes had a high correlation with that of at least one of the nine miRNA markers (Figure 3A). The UpSet plot, generated for visualizing intersecting sets of these differentially expressed genes, showed that one gene might be regulated by up to four miRNA markers (Figure 3B). The KEGG enrichment analyses of these interacted genes revealed various significantly enriched signalling pathways, such as the MAPK signalling pathway, focal adhesion, the Wnt signalling pathway, cell adhesion molecules, the Hedgehog signalling pathway and some pathways involved in cancers (Figure 3C and Table S6).

### 3.4 Specific prognostic miRNA markers of EC patients

**Figure 4:**
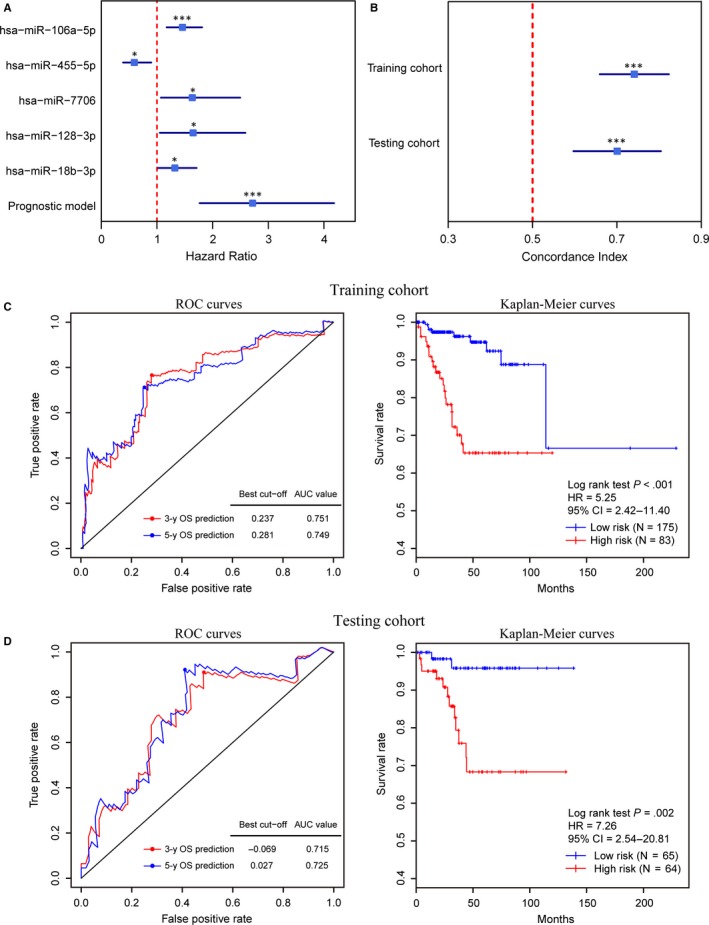
Construction and validation of the prognostic miRNA model. A, Forest plots of hazard ratios of the five miRNA markers and prognostic model in the training cohort. B, Forest plots of the concordance index of the prognostic model in the training and testing cohorts. (*P < .05, **P < .01, ***P < .001) (C, D) Performance of the prognostic model in the OS prediction in the training (C), and testing (D) cohorts. The ROC curves (left) were generated for the 3‐ and 5‐year OS predictions of EC. The best cut‐off value of the 5‐year OS prediction was obtained to divide the patients into low‐ and high‐risk groups. Kaplan‐Meier curves (right) based on these two groups were plotted to analyse the correlations between this model and the OS

Subsequently, the prognostic utility of miRNA signatures for EC was assessed. Only miRNAs with significant differences in expression level between EC and normal endometrium tissues were retained for following analysis. Using the training cohort, miRNAs associated with OS were identified with *P* values less than .05 (Table S7). Through variable screening using the machine learning method and multivariate Cox regression, five specific prognostic miRNA markers, including hsa‐miR‐128‐3p, hsa‐miR‐106a‐5p, hsa‐miR‐7706, hsa‐miR‐18b‐3p and hsa‐miR‐455‐5p, were selected as candidates (Figure 4A).

The expression data of these five markers in the training cohort were collected to establish a prognostic model (Table 3). The concordance indices calculated from the training and testing cohorts were 0.74 (95% CI: 0.66‐0.82) and 0.70 (95% CI: 0.60‐0.80), respectively (Figure 4B). The predictive ability of the five‐miRNA prognostic model was examined in the training and testing cohorts. For the training cohort, the time‐dependent ROC curves for the 3‐ and 5‐year OS prediction were plotted with AUCs of 0.751 and 0.749 (Figure 4C), respectively. The best cut‐off point of the risk score (0.281) was calculated according to the maximum Youden index in the ROC curve for 5‐year survival prediction and was then used to stratify patients into two risk groups (Figure 4C). A significant difference between the OS for patients in these two risk groups was uncovered by the Kaplan‐Meier curve (HR = 5.25, *P* < .001, Figure 4C). A similar situation was found in the testing cohort (Figure 4D). These results suggest that the five‐miRNA model can be used for survival prediction of EC patients.

**Table 3:** The prognostic miRNA model constructed in this study

Marker	MIMATid	Coefficient	Expression level association with poor prognosis
hsa‐miR‐18b‐3p	MIMAT0004751	0.155	High
hsa‐miR‐128‐3p	MIMAT0000424	0.233	High
hsa‐miR‐106a‐5p	MIMAT0000103	0.420	High
hsa‐miR‐455‐5p	MIMAT0003150	−0.495	Low
hsa‐miR‐7706	MIMAT0030021	0.294	High

### 3.5 Correlation of prognostic miRNA markers and their regulated genes

**Figure 5:**
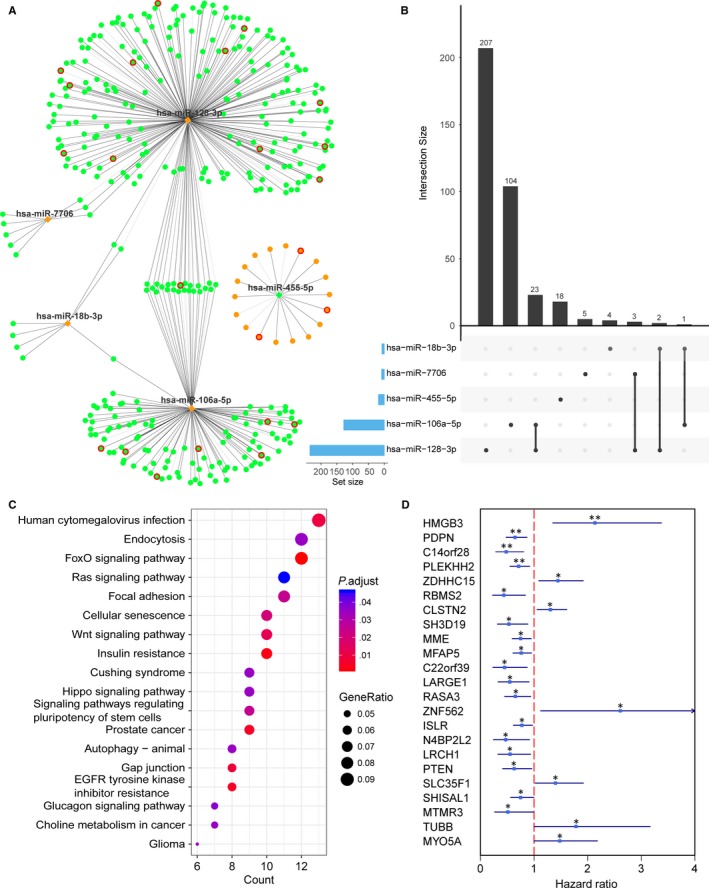
The regulatory network of five miRNA markers and target genes in EC. A, Cytoscape visualization of the 396 miRNA‐mRNA pairs. The diamonds and circles represent the miRNAs and target genes, respectively. The orange and green colours represent up‐regulation and down‐regulation, respectively. The red circle indicates the genes associated with patient survival. The grey edge indicates the verified miRNA‐target pairs, whereas the black edge indicates the predicted pairs. B, The UpSet plot of the interactions between the five prognostic miRNA markers. One miRNA may have up to two regulated genes. C, KEGG functional enrichment analysis for 367 target genes regulated by the five prognostic miRNA markers. Only the pathways with an adjusted *P* value threshold of <.05 were displayed. D, Forest plots of hazard ratios of the 23 genes associated with survival of EC

miRNA‐mRNA expression correlation analysis showed that there were 396 miRNA‐mRNA pairs that were not only negatively correlated but also predicted by algorithms or verified by experiment. The miRNA‐mRNA regulation network is presented in Figure 5A, and a total of 367 genes were identified, whose expressions were highly correlated with these five miRNA markers. Among these regulated genes, one gene might be regulated by multiple miRNA markers (Figure 5B). Kyoto Encyclopedia of Genes and Genomes functional enrichment analysis for these 367 target genes was conducted and showed that these target genes were significantly related to the FoxO signalling pathway, Ras signalling pathway, Wnt signalling pathway etc (Figure 5C and Table S8). It is noteworthy that 23 target genes were found to be associated with the OS of EC (Figure 5D).

### 3.6 Performance assessment of the prognostic miRNA model

**Figure 6:**
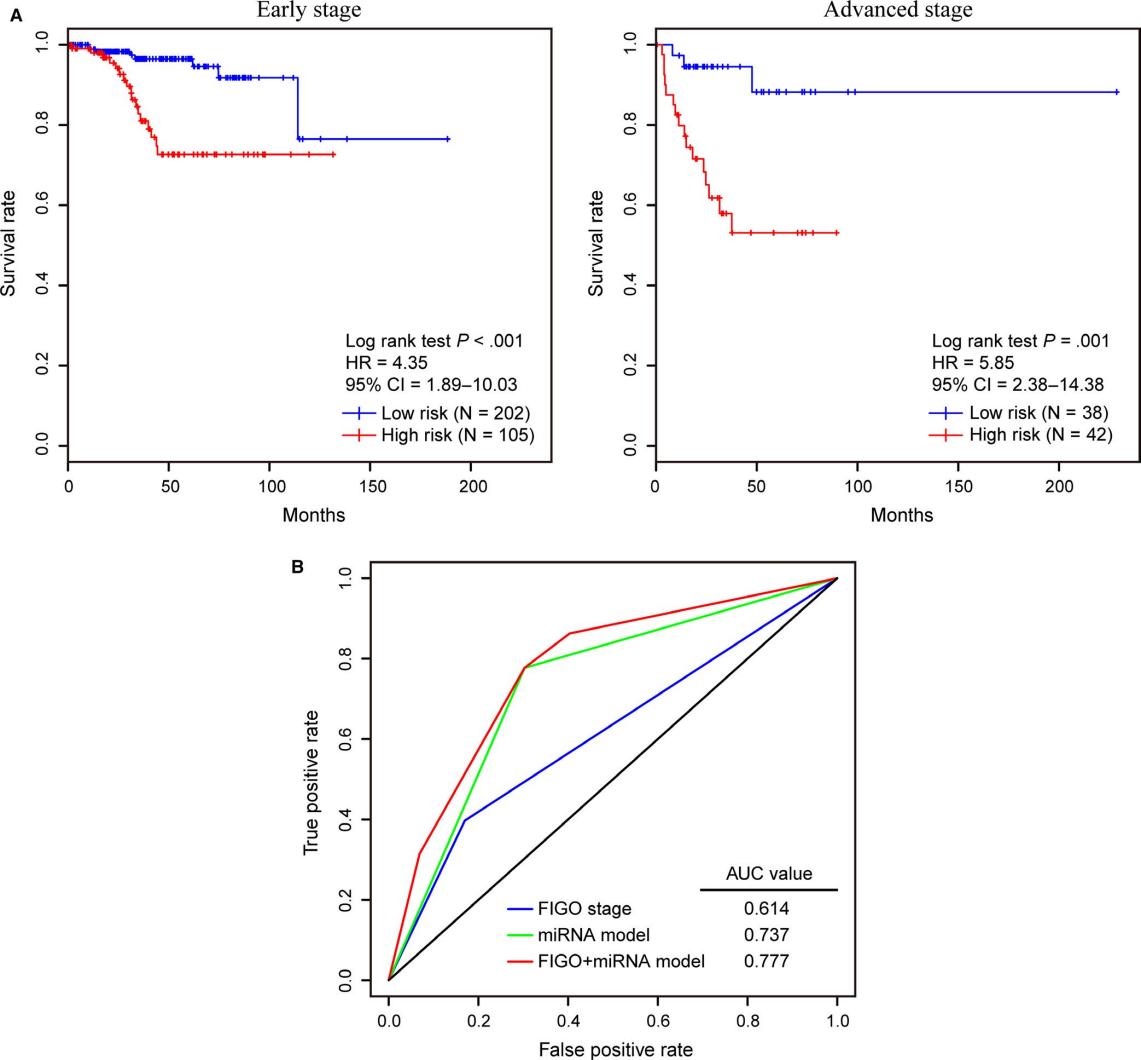
Performance of the miRNA prognostic model in the OS prediction of EC patients stratified by FIGO stage. A, EC patients with early (FIGO I/II stage) and advanced stages (FIGO III/IV stage) were divided into high‐ and low‐risk groups using the miRNA prognostic model, respectively. By plotting Kaplan‐Meier curves, the prognostic model capability for the OS prediction of EC patients with early stage (left) and advanced stage (right) was evaluated individually. B, Comparison of the survival prediction power of the FIGO stage and the prognostic models by ROC curve analysis for the 5‐year OS prediction

To evaluate whether the predictive power of the five‐miRNA model is independent of other clinical factors, we included this model in univariate (Table 4) and multivariate (Table 5) Cox regression analyses together with age, FIGO stage, and histologic grade using training and testing cohorts. The results indicated that both this model and FIGO stage have high prognostic significance (all *P* < .05). Then, to investigate the effectiveness of the prognostic model in patients with different FIGO stages, stratification analysis was conducted based on FIGO stage. As shown in Figure 6A, this miRNA model can divide EC patients in the early stage (FIGO I/II stage) and advanced stage (FIGO III/IV stage) into high‐ and low‐risk groups, respectively. Moreover, this model performed better than FIGO stage (AUC = 0.737 vs AUC = 0.614) and might have a higher prediction power when integrated with the FIGO stage (AUC = 0.777, Figure 6B).

**Table 4:** Univariable Cox regression analysis of potential prognostic variables for EC patients

Clinical features		Training cohort (N = 258)		Testing cohort (N = 129)	
		HR (95% CI)	*P* value	HR (95% CI)	*P* value
miRNA model	High risk vs Low risk	5.29 (2.42‐11.56)	3.04E‐05	7.33 (1.64‐32.82)	9.20E‐03
Age	>60 vs ≤60	1.355 (0.64‐2.86)	.42	3.74 (1.04‐13.42)	4.34E‐02
FIGO stage	Advanced stage vs Early stage	3.09 (1.50‐6.38)	2.21E‐03	3.56 (1.23‐10.25)	1.89E‐02
Histologic grade	G3 vs G1/G2	3.85 (1.71‐8.65)	1.10E‐03	1.81 (0.61‐5.40)	.29

Note

Advanced stage: FIGO I/II stage; Early stage: FIGO III/IV stage.

**Table 5:** Multivariable Cox regression analysis of potential prognostic variables for EC patients

Clinical features		Training cohort (N = 258)		Testing cohort (N = 129)	
		HR (95% CI)	*P* value	HR (95% CI)	*P* value
miRNA model	High risk vs Low risk	3.97 (1.751‐9.02)	9.66E‐04	4.85 (1.02‐22.99)	4.69E‐02
Age	>60 vs ≤60	–	–	3.35 (0.90‐12.43)	.07
FIGO stage	Advanced stage vs Early stage	2.32 (1.10‐4.89)	2.65E‐02	2.97 (0.99‐8.94)	5.22E‐02
Histologic grade	G3 vs G1/G2	2.12 (0.89‐5.05)	.09	–	–

Note

Advanced stage: FIGO I/II stage; Early stage: FIGO III/IV stage. Only significant variables in univariate analysis were included.

## 4 DISCUSSION

Early and accurate diagnosis plays a critical role in the prevention and treatment of cancer. In this study, we explored valuable diagnostic biomarkers associated with EC carcinogenesis by utilizing miRNA expression data. Based on miRNA expression profiles, a total of nine diagnostic miRNA markers were identified that could distinguish EC and normal endometrium tissues, including four putative tumour suppressor miRNAs (hsa‐miR‐152‐3p, hsa‐miR‐24‐1‐5p, hsa‐miR‐374b‐5p and hsa‐miR‐542‐3p) and five oncomiRNAs (hsa‐miR‐1307‐3p, hsa‐miR‐183‐3p, hsa‐miR‐183‐5p, hsa‐miR‐200b‐3p and hsa‐miR‐429) (Table 1). Derived from previous reports, the roles of these miRNAs in carcinogenesis of EC or other cancers have been demonstrated. In support of our findings, miR‐542‐3p is down‐regulated in endometrial serous adenocarcinoma compared to normal endometrial tissues,^19^ and it inhibits tumour angiogenesis and progression via directly targeting the protein angiopoietin‐2.^20^ miR‐152 has been reported to be a tumour suppressor miRNA that is silenced by DNA hypermethylation in EC.^21^ As previously reported, miR‐200b and miR‐429 are oncomiRNAs that target the *PTEN* gene in endometrioid endometrial carcinoma.^22^ MiR‐374b‐5p could suppress tumour progression and enhance cisplatin sensitivity in ovarian cancer by targeting *FOXP1*.^23^ MiR‐1307‐3p has been shown to be overexpressed in breast cancer and could be used as a diagnostic marker.^24^ Noteworthy, miR‐183 was found to be overexpressed in endometrioid endometrial adenocarcinoma,^25^ which is consistent with our finding, whereas another study reported that the up‐regulated expression of miR‐183‐5p promoted apoptosis and suppressed the epithelial‐mesenchymal transition, proliferation, invasion and migration of human endometrial cancer cells.^26^ The role of miR‐183‐5p in EC needs to be further elucidated. Additionally, miR‐24‐1‐5p could act as an oncogene that facilitates the proliferation of ovarian epithelial cells,^27^ which is contrary to our findings. The functional differences in the role of the same miRNA in different cancers might be due to tissue specificity.

Compared with a single biomarker alone, the aggregation of multiple biomarkers into one model can improve the prognostic ability.^28^ Moreover, the establishment of a classifier or model that provided biomarker coefficients or a risk scoring formulas would be beneficial to the wide application of these markers/models in clinical practice.^6^ Therefore, a diagnostic classifier that aggregated the aforementioned miRNA markers was constructed. Performance assessments were also conducted by using a fusion table and ROC curves, which further confirmed the accuracy and effectiveness of this miRNA classifier for EC diagnosis.

We also tried to discover specific biomarkers for predicting prognoses of EC patients based on miRNA expression data. By performing multiple screening procedures, five specific miRNAs were eventually chosen as prognostic markers that were involved in the development and prognosis of EC. Regarding the characteristics of these signature miRNAs, higher expression levels of hsa‐miR‐18b‐3p, hsa‐miR‐128‐3p, hsa‐miR‐106a‐5p and hsa‐miR‐7706 correlated with a shorter OS. For another, higher expression level of hsa‐miR‐455‐5p is associated with a longer OS. Specifically, consistent with our results, miR‐106a was found to be overexpressed in both endometrioid endometrial adenocarcinoma and endometrial serous adenocarcinoma.^7^ Lower expression of miR‐455‐5p is significantly correlated with poor overall survival of endometrial serous adenocarcinoma, and miR‐18b was observed to be overexpressed in endometrial serous adenocarcinoma.^19^ Although there is no EC study on miR‐128‐3p, high expression level of miR‐128‐3p was found in endometriosis plasma and it can be a potential diagnostic biomarker for endometriosis.^29^ Similarly, miR‐7706 has been shown to inhibit the proliferation of hepatocellular carcinoma.^30^ Lymphovascular space invasion (LVSI) is considered as a major determinant of recurrence and overall survival in endometrial carcinomas.^31^ Canlorbe et al^32^ identified three miRNAs (has‐miR‐34c‐5p, has‐miR‐23b‐5p and has‐miR‐23c) associated with LVSI, which may be used as a diagnostic tool for LVSI status. Although these miRNAs were not included in our prognostic model, has‐miR‐23c was shown to be associated with prognosis of EC in our study (*P* < .05, Table S7).

Notably, 23 target genes regulated by prognostic miRNA markers were determined to be associated with the survival of EC patients via correlation analyses. Among these genes, *PDPN*, which is up‐regulated in transformed cells, may be used as a biomarker and therapeutic target for many types of cancer, including glioma, squamous cell carcinoma, mesothelioma and melanoma.^33^ *C14orf28* was reported to be a target of miR‐519d, which contributes to tumorigenesis and might provide new potential targets for colorectal cancer therapy.^34^ In addition, previous studies demonstrated that *MFAP5* could promote tumour progression and bone metastasis by regulating the ERK/MMP signalling pathways in breast cancer.^35^, ^36^ For the *LARGE* gene, previous findings have shown that the silencing of LARGE is responsible for the defects in dystroglycan‐mediated cell adhesion and points to a defect in dystroglycan glycosylation as a factor in cancer progression.^37^ *PTEN*, a tumour suppressor that is mutated in a large number of cancers at high frequency,^38^, ^39^, ^40^, ^41^ is also included in our prognostic models.

A miRNA prognostic model was also constructed based on these five miRNA markers. By plotting ROC curves and Kaplan‐Meier curves, these models exhibited their ability to predict prognoses of EC patients. Further evaluation procedures revealed that this miRNA model was an independent prognostic factor for prognostic prediction of EC patients. Prior to this study, several prognostic models have been developed for EC based on gene signatures. Previously, we have proposed a nine‐gene model for survival prediction in EC, whose AUC values reached 0.82 and 0.676 in the training and validation datasets, respectively.^15^ A six‐gene signature devised by Wang et al^16^ also achieved good performance with AUC values of 0.841 and 0.722. Nevertheless, due to the heterogeneity of validation cohort, it is not appropriate to judge the merits of the model by simply comparing the AUC values in different study. Therefore, we compared our five‐miRNA model with these gene models using our testing cohort, and our model showed an increased AUC value (Figure S1). Moreover, the OS prediction power of this model was compared with that of FIGO stage, further demonstrating that this novel prognostic model has higher accuracy and could assist FIGO stage to predict the prognosis of EC patients. An integrated genomic‐pathologic classification proposed by TCGA study classified EC into four categories: POLE ultramutated, microsatellite instability hypermutated, copy‐number low, and copy‐number high.^3^ And these categories have been shown to be associated with varying degrees of clinical outcome.^3^, ^42^ It is noteworthy that there was a significant association between our prognostic model and this molecular classification (*P* < .01, Table S9), reflecting the prognostic ability of this model. Moreover, given that the extensive genomic characterization undertaken by TCGA is difficult to accomplish in most clinical settings and the translation into clinical application is impractical, the model may also be an alternative or complementary approach to the molecular classification of EC.

To investigate the functional roles of identified miRNA markers, we constructed miRNA‐mRNA regulation networks and identified 375 and 367 genes regulated by diagnostic and prognostic miRNA markers, respectively. Functional enrichment analyses of these targeted genes revealed several potential pathways that might be related to both carcinogenesis and progression of EC. It is noteworthy that some of these pathways have been reported to be linked to cancer. MAPKs regulate various cellular activities related to cancer development, including proliferation, differentiation, apoptosis, inflammation and immunity.^43^ Previous studies found that the crosstalk between MAPK signalling and ER status might exert a key role in the progression of EC.^44^ Wnt signalling, which is pivotal in embryogenesis, healing and homeostasis, is important in the endometrium and has been linked to carcinogenesis.^45^ Current studies have discovered that β‐catenin mutations in Wnt signalling are reported in approximately 20%‐50% of endometrioid endometrial carcinomas.^46^Traditionally, oxytocin (OT) is well known to play an important role in the regulation of cyclic changes in the uterus implantation of embryos and parturition, although oxytocin also is shown as a growth regulator, participating in endothelial cell growth and migration.^47^ Moreover, several experiments have demonstrated that OT signalling serves as a major factor involved in the cell invasion of EC.^48^ The Hippo pathway has been implicated in epithelial‐to‐mesenchymal transition and stemness,^49^ and previous findings revealed a role of the Hippo pathway in the progression of aggressive subtypes of EC.^50^ Aberrant JAK/STAT signalling has been shown to contribute to cancer progression and metastasis,^51^ and targeting the JAK/STAT pathway is currently one of the most promising strategies for prostate cancer treatment. Whereas p53 is the key protein in the pathway and has been considered to play an important role in tumorigenesis, the p53 pathway is one of multiple oncogenic pathways in EC.^52^ Markers of the p53 pathway improve the stratification of EC and provide novel insights into the role of this pathway in the disease.^53^

The limited sample size of EC tissue samples imposes limitations on this study. Therefore, before clinical application, more EC and normal endometrium samples are needed for further validating the diagnostic and prognostic value of the constructed models. Additionally, the mechanisms of most diagnostic and prognostic markers of EC were unclear, and downstream experimental studies on these markers are necessary, which will further deepen the understanding of their functions.

In summary, we identified a series of novel diagnostic miRNA markers and subsequently constructed and validated a diagnostic classifier that could accurately and effectively distinguish between EC and normal endometrium tissues. We also screened prognostic miRNA markers and established a prognostic model that can assist FIGO stage in survival prediction for EC patients. This study also presents novel miRNA therapeutic targets for patients with EC and provides new insights into the mechanisms underlying EC.

## CONFLICT OF INTEREST

The authors declare that there is no conflict of interest.

## AUTHOR CONTRIBUTIONS

JY and DH designed the study and revised the manuscript. QW, KX and YT analysed the data and drafted the manuscript. KX and XD interpreted the results of experiments. TX contributed to the writing of the manuscript. All authors read and approved the final manuscript. QW, KX and YT contributed equally to this work.

## Supporting information

Figure S1

Table S1

Table S2

Table S3

Table S4

Table S5

Table S6

Table S7

Table S8

Table S9

## Data Availability

The data that support the findings of this study are available from the corresponding author upon reasonable request.

## References

[1] Jayaraman M , Radhakrishnan R , Mathews CA , et al. Identification of novel diagnostic and prognostic miRNA signatures in endometrial cancer. Genes Cancer. 2017;8:566‐576. 28740575 10.18632/genesandcancer.144 PMC5511890

[2] Siegel RL , Miller KD , Jemal A . Cancer statistics, 2018. CA: A Cancer J Clin. 2018;68:7‐30. 10.3322/caac.21442 29313949

[3] Cancer Genome Atlas Research Network , Kandoth C , Schultz N , et al. Integrated genomic characterization of endometrial carcinoma. Nature. 2013;497:67‐73. 23636398 10.1038/nature12113 PMC3704730

[4] Yang JY , Werner HM , Li J , et al. Integrative protein‐based prognostic model for early‐stage endometrioid endometrial cancer. Clin Cancer Res. 2016;22:513‐523. 26224872 10.1158/1078-0432.CCR-15-0104 PMC4715969

[5] Wang Q , Xu T , Tong Y , et al. Prognostic potential of alternative splicing markers in endometrial cancer. Mol Ther Nucl Acids. 2019;18:1039‐1048. 10.1016/j.omtn.2019.10.027 PMC6889075 31785579

[6] Ying J , Xu T , Wang Q , Ye J , Lyu J . Exploration of DNA methylation markers for diagnosis and prognosis of patients with endometrial cancer. Epigenetics. 2018;13:490‐504. 29912667 10.1080/15592294.2018.1474071 PMC6140821

[7] Vasilatou D , Sioulas VD , Pappa V , Papageorgiou SG , Vlahos NF . The role of miRNAs in endometrial cancer. Epigenomics. 2015;7:951‐959. 26443384 10.2217/epi.15.41

[8] Bueno MJ , Perez de Castro I , Malumbres M . Control of cell proliferation pathways by microRNAs. Cell Cycle. 2008;7:3143‐3148. 18843198 10.4161/cc.7.20.6833

[9] Torres A , Torres K , Pesci A , et al. Diagnostic and prognostic significance of miRNA signatures in tissues and plasma of endometrioid endometrial carcinoma patients. Int J Cancer. 2013;132:1633‐1645. 22987275 10.1002/ijc.27840

[10] Shih KK , Qin LX , Tanner EJ , et al. A microRNA survival signature (MiSS) for advanced ovarian cancer. Gynecologic Oncol. 2011;121:444‐450. 10.1016/j.ygyno.2011.01.025 21354599

[11] Chen LL , Zhang ZJ , Yi ZB , Li JJ . MicroRNA‐211‐5p suppresses tumour cell proliferation, invasion, migration and metastasis in triple‐negative breast cancer by directly targeting SETBP1. British J Cancer. 2017;117:78‐88. 10.1038/bjc.2017.150 PMC5520212 28571042

[12] Lan H , Lu H , Wang X , Jin H . MicroRNAs as potential biomarkers in cancer: opportunities and challenges. BioMed Res Int. 2015;2015:125094. 25874201 10.1155/2015/125094 PMC4385606

[13] Sun H , Yan L , Tu R , et al. Expression profiles of endometrial carcinoma by integrative analysis of TCGA data. Gynecol Obstet Invest. 2017;82:30‐38. 26986489 10.1159/000445073

[14] Love MI , Huber W , Anders S . Moderated estimation of fold change and dispersion for RNA‐seq data with DESeq2. Genome Biol. 2014;15:550. 25516281 10.1186/s13059-014-0550-8 PMC4302049

[15] Ying J , Wang Q , Xu T , Lyu J . Establishment of a nine‐gene prognostic model for predicting overall survival of patients with endometrial carcinoma. Cancer Med. 2018;7:2601‐2611. 29665298 10.1002/cam4.1498 PMC6010780

[16] Wang Y , Ren F , Chen P , Liu S , Song Z , Ma X . Identification of a six‐gene signature with prognostic value for patients with endometrial carcinoma. Cancer Med. 2018;7:5632‐5642. 30306731 10.1002/cam4.1806 PMC6247034

[17] Dweep H , Gretz N . miRWalk2.0: a comprehensive atlas of microRNA‐target interactions. Nature Methods. 2015;12:697. 26226356 10.1038/nmeth.3485

[18] Yu G , Wang LG , Han Y , He QY . clusterProfiler: an R package for comparing biological themes among gene clusters. Omics. 2012;16:284‐287. 22455463 10.1089/omi.2011.0118 PMC3339379

[19] Hiroki E , Akahira J , Suzuki F , et al. Changes in microRNA expression levels correlate with clinicopathological features and prognoses in endometrial serous adenocarcinomas. Cancer Sci. 2010;101:241‐249. 19891660 10.1111/j.1349-7006.2009.01385.x PMC11159282

[20] He T , Qi F , Jia L , et al. MicroRNA‐542‐3p inhibits tumour angiogenesis by targeting angiopoietin‐2. J Pathol. 2014;232:499‐508. 24403060 10.1002/path.4324

[21] Tsuruta T , Kozaki K , Uesugi A , et al. miR‐152 is a tumor suppressor microRNA that is silenced by DNA hypermethylation in endometrial cancer. Cancer Res. 2011;71:6450‐6462. 21868754 10.1158/0008-5472.CAN-11-0364

[22] Yoneyama K , Ishibashi O , Kawase R , Kurose K , Takeshita T . miR‐200a, miR‐200b and miR‐429 are onco‐miRs that target the PTEN gene in endometrioid endometrial carcinoma. Anticancer Res. 2015;35:1401‐1410. 25750291

[23] Li H , Liang J , Qin F , Zhai Y . MiR‐374b‐5p‐FOXP1 feedback loop regulates cell migration, epithelial‐mesenchymal transition and chemosensitivity in ovarian cancer. Biochem Biophys Res Commun. 2018;505:554‐560. 30274777 10.1016/j.bbrc.2018.09.161

[24] Shimomura A , Shiino S , Kawauchi J , et al. Novel combination of serum microRNA for detecting breast cancer in the early stage. Cancer Sci. 2016;107:326‐334. 26749252 10.1111/cas.12880 PMC4814263

[25] Chung TK , Cheung TH , Huen NY , et al. Dysregulated microRNAs and their predicted targets associated with endometrioid endometrial adenocarcinoma in Hong Kong women. Int J Cancer. 2009;124:1358‐1365. 19065659 10.1002/ijc.24071 PMC6953413

[26] Yan H , Sun BM , Zhang YY , et al. Upregulation of miR‐183‐5p is responsible for the promotion of apoptosis and inhibition of the epithelial‐mesenchymal transition, proliferation, invasion and migration of human endometrial cancer cells by downregulating Ezrin. Int J Mol Med. 2018;42:2469‐2480. 30226564 10.3892/ijmm.2018.3853 PMC6192766

[27] Zhang W , Fei J , Yu S , et al. LINC01088 inhibits tumorigenesis of ovarian epithelial cells by targeting miR‐24‐1‐5p. Sci Rep. 2018;8:2876. 29440672 10.1038/s41598-018-21164-9 PMC5811426

[28] Kratz JR , He J , Van Den Eeden SK , et al. A practical molecular assay to predict survival in resected non‐squamous, non‐small‐cell lung cancer: development and international validation studies. Lancet. 2012;379:823‐832. 22285053 10.1016/S0140-6736(11)61941-7 PMC3294002

[29] Zhuo Z , Wang C , Li G , Yu H . Plasma MicroRNAs can be a potential diagnostic biomarker for endometriosis. Preprints. 2019 10.20944/preprints201907.0108.v1 34155616

[30] Wang F , Dai M , Chen H , et al. Prognostic value of hsa‐mir‐299 and hsa‐mir‐7706 in hepatocellular carcinoma. Oncology letters. 2018;16:815‐820. 29963149 10.3892/ol.2018.8710 PMC6019942

[31] Bendifallah S , Canlorbe G , Raimond E , et al. A clue towards improving the European Society of Medical Oncology risk group classification in apparent early stage endometrial cancer? Impact of lymphovascular space invasion. British J Cancer. 2014;110:2640‐2646. 10.1038/bjc.2014.237 PMC4037837 24809776

[32] Canlorbe G , Castela M , Bendifallah S , et al. Micro‐RNA signature of lymphovascular space involvement in type 1 endometrial cancer. Histology Histopathol. 2017;32:941‐950. 10.14670/HH-11-859 28000202

[33] Krishnan H , Rayes J , Miyashita T , et al. Podoplanin: an emerging cancer biomarker and therapeutic target. Cancer Sci. 2018;109:1292‐1299. 29575529 10.1111/cas.13580 PMC5980289

[34] Yang X , Hu Y , Liu Y , et al. C14orf28 downregulated by miR‐519d contributes to oncogenicity and regulates apoptosis and EMT in colorectal cancer. Molecular Cellular Biochem. 2017;434:197‐208. 10.1007/s11010-017-3049-2 28455792

[35] Mok SC , Bonome T , Vathipadiekal V , et al. A gene signature predictive for outcome in advanced ovarian cancer identifies a survival factor: microfibril‐associated glycoprotein 2. Cancer Cell. 2009;16:521‐532. 19962670 10.1016/j.ccr.2009.10.018 PMC3008560

[36] Wu Z , Wang T , Fang M , et al. MFAP5 promotes tumor progression and bone metastasis by regulating ERK/MMP signaling pathways in breast cancer. Biochem Biophys Res Commun. 2018;498:495‐501. 29526753 10.1016/j.bbrc.2018.03.007

[37] de Bernabe DB , Inamori K , Yoshida‐Moriguchi T , et al. Loss of alpha‐dystroglycan laminin binding in epithelium‐derived cancers is caused by silencing of LARGE. J Biol Chem. 2009;284:11279‐11284. 19244252 10.1074/jbc.C900007200 PMC2670132

[38] Carracedo A , Alimonti A , Pandolfi PP . PTEN level in tumor suppression: how much is too little? Cancer Res. 2011;71:629‐633. 21266353 10.1158/0008-5472.CAN-10-2488 PMC3249925

[39] Jamaspishvili T , Berman DM , Ross AE , et al. Clinical implications of PTEN loss in prostate cancer. Nat Rev Urol. 2018;15:222‐234. 29460925 10.1038/nrurol.2018.9 PMC7472658

[40] Knobbe CB , Merlo A , Reifenberger G . Pten signaling in gliomas. Neuro‐Oncol. 2002;4:196‐211. 12084351 PMC1920635

[41] Wallace JA , Li F , Leone G , Ostrowski MC . Pten in the breast tumor microenvironment: modeling tumor‐stroma coevolution. Cancer Res. 2011;71:1203‐1207. 21303970 10.1158/0008-5472.CAN-10-3263 PMC3075554

[42] Cosgrove CM , Tritchler DL , Cohn DE , et al. An NRG Oncology/GOG study of molecular classification for risk prediction in endometrioid endometrial cancer. Gynecol Oncol. 2018;148:174‐180. 29132872 10.1016/j.ygyno.2017.10.037 PMC5756518

[43] Kim EK , Choi EJ . Compromised MAPK signaling in human diseases: an update. Archives Toxicol. 2015;89:867‐882. 10.1007/s00204-015-1472-2 25690731

[44] Zhou L , Cai B , Bao W , et al. Crosstalk between estrogen receptor and mitogen‐activated protein kinase signaling in the development and progression of endometrial cancer. Int J Gynecol Cancer. 2011;21:1357‐1365. 21720253 10.1097/IGC.0b013e3182216ac9

[45] Coopes A , Henry CE , Llamosas E , Ford CE . An update of Wnt signalling in endometrial cancer and its potential as a therapeutic target. Endocr‐Relat Cancer. 2018;25:647‐662.. 10.1530/ERC-18-0112 30093601

[46] Ikeda T , Yoshinaga K , Semba S , Kondo E , Ohmori H , Horii A . Mutational analysis of the CTNNB1 (beta‐catenin) gene in human endometrial cancer: frequent mutations at codon 34 that cause nuclear accumulation. Oncol Rep. 2000;7:323‐326. 10671680 10.3892/or.7.2.323

[47] Cassoni P , Marrocco T , Bussolati B , et al. Oxytocin induces proliferation and migration in immortalized human dermal microvascular endothelial cells and human breast tumor‐derived endothelial cells. Mol Cancer Res: MCR. 2006;4:351‐359. 16778082 10.1158/1541-7786.MCR-06-0024

[48] Dery MC , Chaudhry P , Leblanc V , Parent S , Fortier AM , Asselin E . Oxytocin increases invasive properties of endometrial cancer cells through phosphatidylinositol 3‐kinase/AKT‐dependent up‐regulation of cyclooxygenase‐1, ‐2, and X‐linked inhibitor of apoptosis protein. Biol Reprod. 2011;85:1133‐1142. 21816851 10.1095/biolreprod.111.093278 PMC4480429

[49] Moroishi T , Hansen CG , Guan KL . The emerging roles of YAP and TAZ in cancer. Nat Rev Cancer. 2015;15:73‐79. 25592648 10.1038/nrc3876 PMC4562315

[50] Romero‐Perez L , Garcia‐Sanz P , Mota A , et al. A role for the transducer of the Hippo pathway, TAZ, in the development of aggressive types of endometrial cancer. Mod Pathol. 2015;28:1492‐1503. 26381823 10.1038/modpathol.2015.102

[51] Pencik J , Pham HT , Schmoellerl J , et al. JAK‐STAT signaling in cancer: from cytokines to non‐coding genome. Cytokine. 2016;87:26‐36. 27349799 10.1016/j.cyto.2016.06.017 PMC6059362

[52] Ito K , Watanabe K , Nasim S , et al. Prognostic significance of p53 overexpression in endometrial cancer. Cancer Res. 1994;54:4667‐4670. 8062261

[53] Edmondson RJ , Crosbie EJ , Nickkho‐Amiry M , et al. Markers of the p53 pathway further refine molecular profiling in high‐risk endometrial cancer: a TransPORTEC initiative. Gynecol Oncol. 2017;146:327‐333. 28511869 10.1016/j.ygyno.2017.05.014

