# Supplementary figures and images for: Novel miRNA markers for the diagnosis and prognosis of endometrial cancer
Source: J Cell Mol Med. 2020 Mar 9;24(8):4533–46. doi: 10.1111/jcmm.15111 (PMC7176884; doi:10.1111/jcmm.15111)

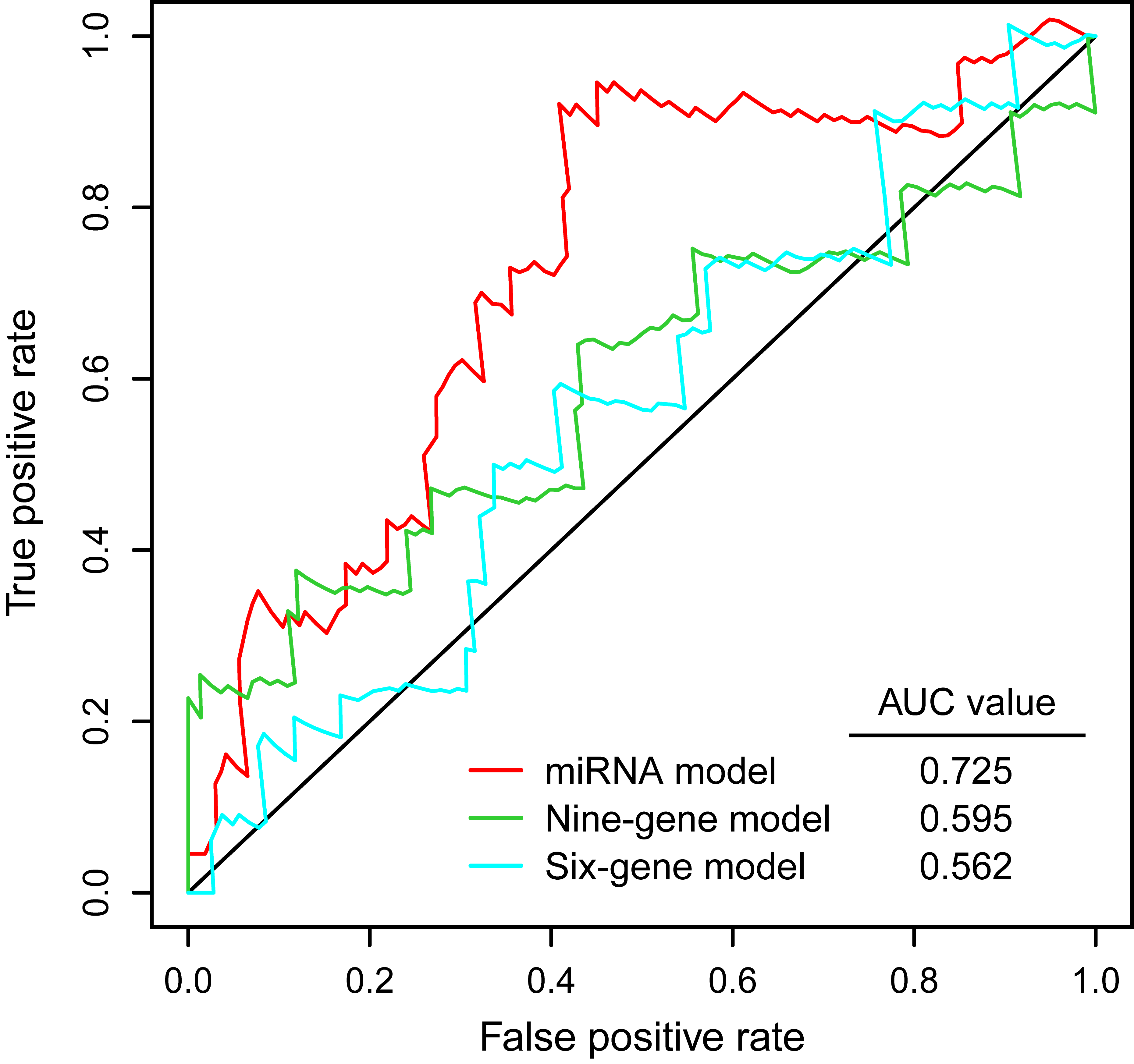

Supplement: Supplementary file 1 — Figure S1 [file JCMM-24-4533-s001.tif]
